# Assessment of Force Retention between Milled Metallic and Ceramic Telescopic Crowns with Different Taper Angles Used for Oral Rehabilitation

**DOI:** 10.3390/ma13214814

**Published:** 2020-10-28

**Authors:** Caroline Adela Ingrid Fischer, Doina Lucia Ghergic, Diana Maria Vranceanu, Stefan Alexandru Ilas, Raluca Monica Comaneanu, Florin Baciu, Cosmin Mihai Cotrut

**Affiliations:** 1 Organizing Institution of University Doctoral Studies, Doctoral School of Dentistry, “Titu Maiorescu” University, 67A Gh. Petrascu Street, 040441 Bucharest, Romania; caroline.fischer@ymail.com; 2 Faculty of Dental Medicine, “Titu Maiorescu” University, 67A Gh. Petrascu Street, 040441 Bucharest, Romania; doinaghergic@yahoo.com (D.L.G.); monica_tarcolea@yahoo.co.uk (R.M.C.); 3 Department of Metallic Materials Science, Physical Metallurgy, Faculty of Materials Science and Engineering, University POLITEHNICA of Bucharest, 313 Splaiul Independentei, 060042 Bucharest, Romania; ilas.stefan.alexandru@gmail.com; 4 Department of Strength Materials, Faculty of Industrial Engineering and Robotics, University POLITEHNICA of Bucharest, 313 Splaiul Independentei, 060042 Bucharest, Romania; florin.baciu@upb.ro

**Keywords:** telescopic crowns, cobalt chromium alloy, zirconia, retention forces, computer-aided design/computer-aided manufacturing (CAD/CAM)

## Abstract

The present study assessed the retention forces corresponding to different telescopic systems used in removable prosthetic dentures. The telescopic systems were represented by Co–Cr alloy or zirconia-based primary crowns and Co–Cr secondary crowns. All crowns were manufactured using computer-aided design/computer-aided manufacturing technology (CAD/CAM). Two types of reference abutment teeth (upper canine and first upper molar) were selected in order to obtain the telescopic crowns and two taper angles—of 0° and 2°—were used for the design of the crowns. A number of 120 samples of telescopic crowns were obtained and subjected to mechanical tests, following a specific protocol, on a mechanical testing equipment. The retention of the telescopic systems was evaluated for different sets of cycles (up to 360), represented by movements that simulate the intraoral insertion and disinsertion of the telescopic systems. The present study highlights that the telescopic systems in which the primary crown is made of zirconia ceramics presents more advantages than those made of Co–Cr. All telescopic systems studied, highlighted that by modifying the taper angle from 0° to 2°, the retention forces have decreased, irrespective of the materials used for the fabrication of the primary crown, suggesting that by using a taper angle of 0°, which is known to be ideal, more efficient, and reliable prosthesis can be developed. Thus, even though the ceramic–metallic telescopic system exhibited the highest retention, all telescopic crowns evaluated registered values between 2–7 N, indicating that they are suitable for clinical use.

## 1. Introduction

Telescopic systems are used to retain removable dentures that are usually recommended for patients with few residual teeth—or who have lost their teeth due to periodontal disease, to patients who have undergone surgical treatment (i.e., for craniofacial malignant tumors), or in cases of occlusal vertical dimension reduction caused by the pathological wear of the teeth [1,2,3]. This system is considered an ideal treatment option when fixed prosthetic treatment cannot be applied due to a compromised, unfavorable general medical condition [4,5]. 

The classic telescopic system is made of two separate elements, consisting of the primary crown—permanently cemented on the prepared abutment tooth—and the secondary crown—that is placed on the mucosal aspect of a removable prosthetic denture [6,7,8,9,10,11,12]. Telescopic systems transfer forces along the long axis of the teeth and provide guidance, support, and protection against the movements that dislocates the denture [6,13]. 

The materials used for crowns manufacturing are gold, titanium, titanium alloys, cobalt–chromium alloys, zirconium-based ceramics, and polymers (especially in the case of three-element systems [14,15,16,17,18,19,20,21,22,23,24,25]). These are materials with different friction coefficients and hardness [26,27,28]. The components of a telescopic crown can be made of the same material or of different ones, which leads to the formation of friction nodes with different tribological features [29].

Because of their excellent mechanical properties and high resistance to corrosion and wear, Co–Cr alloys have been recognized as effective metallic biomaterials for dental applications and medical devices [30,31].

Co–Cr alloys pose good castability and in dental application are usually used in cast solid-state, the microstructure of which exhibits a dendritic α-fcc (face-centered cubic) metastable matrix due to the sluggish nature of the fcc → hcp (hexagonal close packed) transformation and a precipitate, which consists in a M_23_C_6_ carbides, an intermetallic σ compound and a lamellar phase formed by interlayed plates of M_23_C_6_ carbide [32]. The hardness of the alloy in the cast state is mainly due to carbide precipitation at grain boundaries and interdendritic regions but also by dislocation interactions with stacking fault intersections. Alloying elements such as Mo, W, and Si are used for Co–Cr alloys, act as biomaterials due to increasing the hcp-to-fcc transformation temperature, and are also hcp stabilizers. This martensitic transformation is closely related to the microstructure and mechanical and chemical properties of the Co–Cr alloys [31].

Even though Co–Cr alloys present a higher corrosion resistance (resistant to pitting and crevice corrosion) and tarnish but also a good biocompatibility, in recent years, some studies demonstrated that Co–Cr alloys could induce cytotoxicity and inflammatory responses in human gingival fibroblasts and osteoblasts [33,34]. Even so, in the Global Metal Implants and Medical Alloys Market Size, Share and Trends Analysis Report made by Orion Market Research the Co–Cr alloys market alongside with stainless steel and titanium is estimated to grow at a significant Compound Annual Growth Rate (CAGR) of 7.6% during the forecast period (2019–2025). 

Thus, one aim could be the development of biomaterials strong enough to replace the metallic ones. Zirconia-based ceramic has been introduced and used in medicine as an alternative for aluminum- or titanium-based alloy, due to its impressive mechanical properties [35]. In the ceramic classification, zirconia (ZrO_2_) is a highly resistant, polycrystalline ceramic, characterized by favorable mechanical properties, good optical characteristics [23,36,37], aesthetic appearance, and chemical resistance, which are perfectly combined with outstanding biocompatibility proven by clinical studies. In terms of biocompatibility, it was already demonstrated that zirconia has excellent behavior both in vitro and in vivo, lower plaque retention, and good radiopacity, that are perfectly combined with its bioinert character—it is not soluble in water, and its susceptibility to degradation in the harsh, oral environment is neglectable [23,36,37,38]. Moreover, the physical properties of zirconia can be enhanced by stabilization of zirconia with yttrium oxide [39], which is a result of the crystalline modification from a tetragonal to monoclinic arrangement [40]. As mentioned in the literature, zirconia was also used as material for telescopic crown systems [41,42,43] as either primary or secondary crown, or even both. 

The components of a telescopic crown system can be manufactured using classical or unconventional technologies (e.g., casting, computer-aided manufacturing technology, or additive manufacturing techniques), resulting in differences in surface roughness and dimensional tolerances. Computer-aided design and computer-aided manufacturing technologies (CAD/CAM) have greatly increased and diversified the treatment options available to clinicians by being able to produce very complex forms and by the simplicity of their use [44,45,46,47,48,49]. In this context, recent developments in 3D printing have already impacted the field of medicine, including the manufacturing of components that are included in telescopic crown systems [50].

A distinctive feature of telescoping systems, often used to describe and compare them, is the retention force, which is known as a force necessary to separate the two components. Crown retention depends on many factors that can be attributed to the following general categories: materials used for manufacturing technology, working conditions, or measurement method.

With respect to the clinical situation, the retention force can be adjusted by modifying the design. The cross-sectional configuration, conical angle, and contact surface determines the quality and amount of friction between surfaces, which ultimately controls the retention force. Generally, the retention forces of telescopic systems recorded in the scientific literature are between 2–7 N [51,52,53]. One of the most important factors for the success of double-crown systems is setting the optimum retentive force, which requires technical skill, ability, and experience [7,54,55].

The stability of the denture during use is ensured by rigid primary telescopes with cylindrical or conical shapes and a precise match with the secondary telescopes. The narrow configuration between the contact walls generates a compressive tension between the surfaces [4]. The tension should be strong enough to hold the prosthesis in its place. An increase in wall inclination reduces retention between them. The lower the degree of conicity, the greater the friction between the inner and the outer crown. If the abutment height is reduced, either the contact walls must be kept parallel or the degree of conicity of the wall must be modified to improve retention.

One of the most common materials used for telescopic retainers are represented by metallic alloys [56,57,58]; gold alloys are frequently utilized for telescopic crown manufacturing, but their clinical application is limited because of high costs and contraindication for certain patients with metal allergies.

Previous studies [59,60] indicate that a stable and retentive telescopic system can be achieved more easily using the CAD/CAM technology and zirconia (yttria-stabilized tetragonal zirconia polycrystal) as a material of choice. Thus, compared with the conventional manufacturing of metallic telescopic systems, the CAD/CAM technology improves workflow efficiency and reduces technical errors.

In addition, in the literature studied, the retentive forces between primary and secondary telescopic crowns were measured with and without lubrication by a saliva substitute. Bayer et al. [61] stated that no statistically significant differences between lubricated and unlubricated samples were found.

The main purpose of this study was to analyze, by means of specific mechanical tests, the retention forces corresponding to different telescopic systems; these systems were represented by cobalt–chromium (Co–Cr) alloy or zirconia-based primary crowns and Co–Cr secondary crowns. Relevant aspects regarding the evolution of the retention forces of the selected telescopic systems in a dry environment for different set of mechanical cycles at different time periods were pointed out. Additionally, an identification of contact areas between the primary and secondary crowns (displayed after the applied tests) was made in order to highlight the behavior of the selected telescopic systems. 

## 2. Materials and Methods 

The telescopic systems were manufactured by CAD/CAM procedures in a local dental laboratory (Fischer Dental Laboratory, Bucharest, Romania) by experienced dental technicians. The materials used for the manufacturing of the crowns were zirconia (ZrO_2_) and cobalt–chromium (Co–Cr) alloy, from NOVADENT (Hamburg, Germany) in the form of discs. A total of 120 telescopic systems were used. In the present study, the telescopic systems were designed for two reference abutment teeth, specially prepared for telescopic systems: an upper right canine (tooth no. 1.3) and a first upper left molar (tooth no 2.6); besides, the crowns design included two taper angles, 0° and, respectively, 2°. 

CAD–CAM technologies used for manufacturing the telescopic system components required in oral rehabilitation have led to primary and secondary crowns without deviating from the geometry required and dictated by the anatomical shape of the teeth studied.

**Figure 1:**
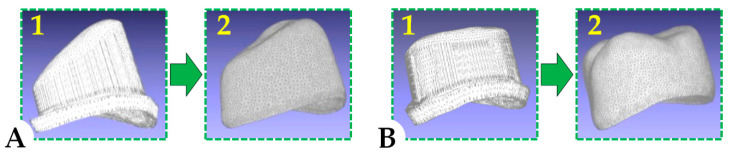
Three-dimensional frames representing the computerized design for the primary (1) and the secondary (2) telescopic crowns dedicated to an upper canine (**A**) and an upper molar (**B**).

The computer-aided design (CAD) stage involves the virtual scanning and modelling of the future prosthetic work. Scanning the model for the clinical situation on which this study was based was performed with a D850 Scanner (3Shape, Copenhagen, Denmark). A schematic illustration of relevant 3D frames representing CAD design for the primary and the secondary telescopic crowns is presented in Figure 1.

Computer-aided manufacturing (CAM) involves the transformation of the previously obtained virtual work into the physical product. For this transformation, the Millbox Software (CIM Systems, Pulheim, Germany) and CORiTEC-350i Milling Machine (Imes-Icore, Eiterfeld, Germany) were used. 

After obtaining the primary and secondary crowns, all samples were investigated in terms of elemental composition and surface morphology; also, a visual inspection of the crowns was made (naked eye and using magnifying glass/×3.5).

The telescopic systems obtained were divided into 8 groups with the following variables: (1) the material used for fabricating the primary crown (Co–Cr alloy or zirconia); (2) the shape of the abutment tooth, canine (1.3) or molar (2.6) and (3) the taper angle of 0° or 2°. 

Each group of 15 telescopic systems was subjected to simulations for five different intervals. The applied mechanical tests were planned to simulate the intraoral detaching and recoupling cycles of the telescopic crowns. These cycles are encountered during daily use, after mounting the telescopic systems in the prosthetic restorations. The systems were cycled for 30, 90, 180, 270, and 360 cycles, which correspond to periods of 1, 3, 6, 9, and 12 months. It was estimated that a patient performs one such cycle per day.

The measurements were achieved in triplicates and the average was calculated. The values obtained were registered, and the retention forces were extracted from the obtained data. The value of retention forces for the first cycle were extracted from the values of the first tests included in the set of 30 cycles (the first value upon the detachment of the secondary crown). 

**Figure 2:**
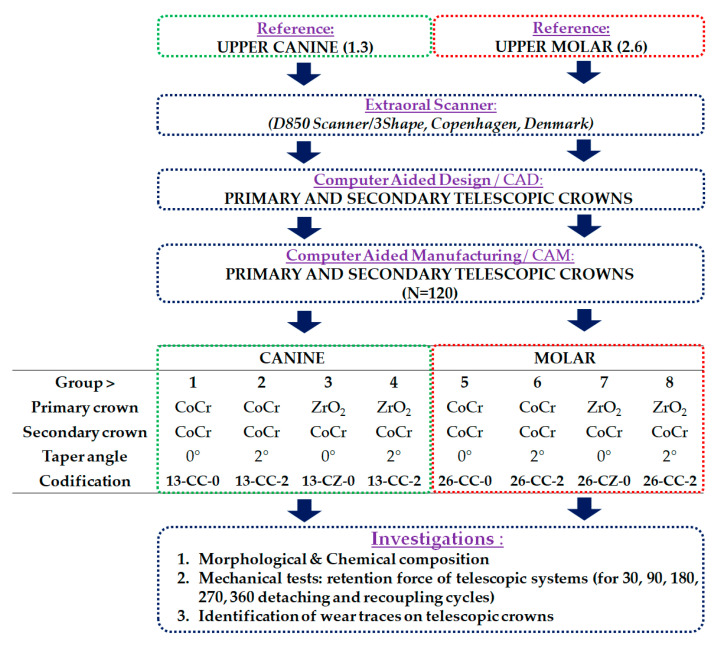
Research design, codification, and characteristic of the telescopic crowns obtained.

The main aspects of the research design and the codification of telescopic system groups are presented in Figure 2 and Table 1.

Before being mechanically tested, the telescopic systems underwent a preparatory stage, which consisted of attaching the primary and secondary crown with copper rods of 2.5 mm in diameter and epoxy resin for fixation. To avoid unwanted loads during mechanical tests and to obtain accurate retention forces values, a special attention has been directed towards the positioning of the rods, which needed to be on the same axis. The materials used to prepare the crowns for mechanical testing (copper rods and epoxy resin) were carefully selected in the experimental design. The tensile strength for both copper rods (33 MPa) and epoxy resin, respectively, (40–60 MPa) have values that will not induce errors during the mechanical tests due to deformation of copper rods or crack failure of epoxy resin. Thus, the cooper rods and epoxy resin behave as rigid bodies during mechanical testing.

The morphology and elemental composition were investigated using a scanning electron microscope (SEM) equipped with an energy dispersion spectrometer (EDS; Phenom ProX, Phenom World, Eindhoven, The Netherlands). SEM images were acquired at 15 kV acceleration voltage using a backscattered electron detector (BSE). Since zirconia is a non-conductive material, a charge reduction sample holder (Phenom ProX, Phenom World, Eindhoven, The Netherlands) was employed for the acquisition of images.

**Figure 3:**
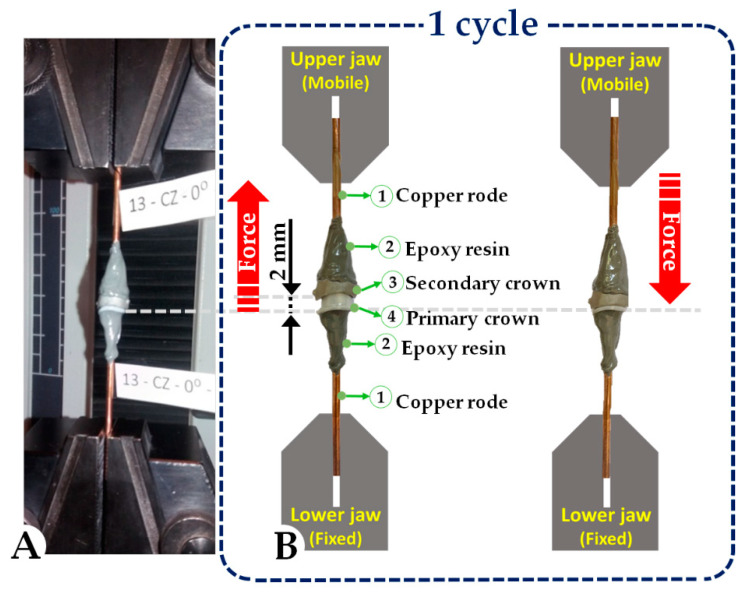
Image of the mechanical testing set-up (**A**) and schematic representation of 1 cycle (**B**).

As there is no standard protocol for in vitro studies in which double-crown systems can be evaluated, the method proposed and used in this study was chosen with great care. Clinically, the secondary crown is fixed on the primary crown through the chewing forces. The chewing force value can vary for each person with respect to the clinical situation such as opposite arch situation, tooth location in relation to the occlusion, etc. [62]. Mechanical tests were performed using a Lloyd LRX Plus (Ametek, Largo, FL, USA) testing equipment. Each sample was individually tested, and the sample rods were positioned into the Lloyd LRX Plus jaws in a vertical position (Figure 3).

The experimental tests applied to the crowns obtained were projected to mimic the conventional insertion and disinsertion of a removable prosthetic denture with telescopic systems (one cycle corresponded to one attaching–detaching movement). As one cycle was supposed to correspond to one day, force retention measurements were made for different time intervals, as follows: 30 cycles (representing one month), 90 cycles (representing 3 months), 180 cycles (representing 6 months), 270 cycles (representing 9 months), and 360 cycles (representing 12 months). 

To be able to record the forces, the movement of the telescopes was set to 2 mm at a speed of 18 cycles/min. During the tests, the forces were recorded with the NEXYGEN Plus software (Ametek, Largo, FL, USA). The software allows parameters, such as the number of cycles, the displacement, and the speed at which all the cycles were performed, to be accurately set in respect of the research design. 

Retention was evaluated by measuring the tensile force required to dislodge the primary crowns from the secondary crown in the universal testing machine. Thus, the extracted values were those of the retention forces recorded at the end of the set number of predetermined cycles. Retention forces were carefully recorded and, as previously stated, for every group tested (8 groups/15 samples per group), for all the different set of cycles, the calculated average values were taken into consideration. 

Additionally, in order to study the wear of the crowns surfaces that are in contact with each other, it was necessary to investigate the telescopic systems after they were obtained, through macroscopic analysis. After the samples were subjected to mechanical tests, they were again macroscopically examined (visual inspection of the crown surfaces: naked eye and using magnifying glass/×3.5), to identify any wear traces that appeared after the cycles were performed. The macroscopic images obtained after the mechanical tests were compared with those obtained initially, in order to observe the differences arising from the wear process.

## 3. Results and Discussion 

### 3.1. Morphology and Chemical Composition

**Figure 4:**
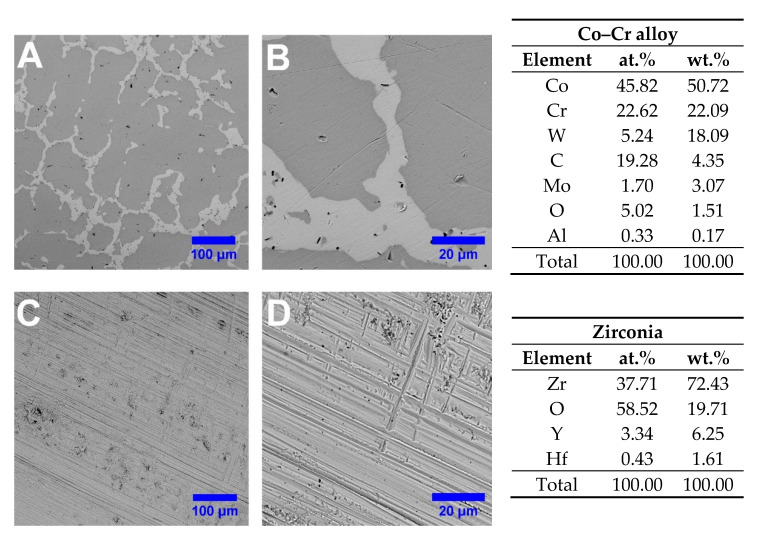
SEM images of a (**A**,**B**) Co–Cr and (**C**,**D**) ZrO_2_ outer surface of primary telescopic crowns along with the corresponding elemental composition.

Initially, the telescopic systems were investigated in terms of elemental composition and surface morphology by SEM–EDS. According to the SEM images achieved on the outer surface of the primary telescopic crowns, on Co–Cr crowns the typical casted solid state morphological features with just a few thin scratches were noticed, while denser and more pronounced scratches for ZrO_2_ were observed, as presented in Figure 4.

After the Co–Cr crowns were obtained by CAM process, a dental technician, in order to accurately reproduce the technological steps met in the clinical practice, resulting in a grazed and smooth surface, polished the outer surfaces. Therefore, as it can be seen, the microstructure consisted of a dendritic matrix with precipitates at grain boundaries and interdendritic regions, which are in good agreement with the results reported in the literature for casted Co–Cr alloys [63]. Thus, it can be said that the milling process has not induced any microstructural modifications in the Co–Cr that might affect or influence the material behavior under specific working conditions.

In the case of ZrO_2_ primary crown after the milling process, the outer surfaces remained as such, even though some thin scratches were noted on the material surface, which are a mark of the CAM process. The chemical compositions obtained by EDS analysis of the Co–Cr and ZrO_2_ materials used to fabricate the telescopic crowns are presented in Figure 4 and were achieved on an area of 90 × 90 µm^2^.

The elemental composition has evidenced the elements corresponding to Co–Cr alloy (Co, Cr, W, Mo, Al) as mentioned by the manufacturer in the technical datasheet, and yttrium-stabilized zirconia ceramic (Zr, O, Y, and Hf). 

**Figure 5:**
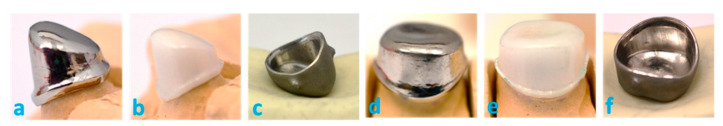
Macroscopic images of the manufactured telescopic system: primary crown made from CoCr (**a**), ZrO_2_ (**b**), and secondary crown (**c**) of tooth 1.3 and primary crown made from CoCr (**d**), ZrO_2_ (**e**), and secondary crown (**f**) of tooth 2.6.

Visual inspection of the crown surfaces (naked eye and using magnifying glass/×3.5) revealed no defects of the telescopic system crowns, except for some green color traces from the post-processing verification process carried out in the dental laboratory by a dental technician that has marked certain areas that required additional processing on some of the samples (Figure 5). Thus, the sets of telescopic systems did not show manufacturing defects.

### 3.2. Mechanical Tests—Retention Forces

A total of 120 telescopic systems divided into eight groups were evaluated in terms of retention forces by mechanical tests. Each group contained 15 samples, and for each time interval, three samples were attributed. The results obtained were exported and calculations were made to determine the mean values of the retention forces and their standard deviations (Table 2).

After the mechanical tests, the values of the forces of detaching the crowns from each other indicated that the component materials, the angle of the primary crown walls, but also the telescopic geometry dictated by the anatomical shape of the teeth analyzed have a strong influence on the retention forces.

Analyzing the results of the retention forces recorded, it can be said that many of them meet the minimal necessary values dictated by the clinical needs [51,52,53,64]. Moreover, according to a study achieved by Stančić and Jelenković [65], the retention forces registered on a canine’s specimen is 6.5 N, while for molars, it is 3.5 N.

In this context, recent studies [64] highlight the advances of artificial intelligence and machine learning in the simulation of 3D bio-prosthetic printing, their impact on safe design, prediction, evaluation, and manufacturing of future generations of medical products. The evolution of technology allows large perspectives for the development of new materials, more performing ones, including in the domain of dental medicine.

**Figure 6:**
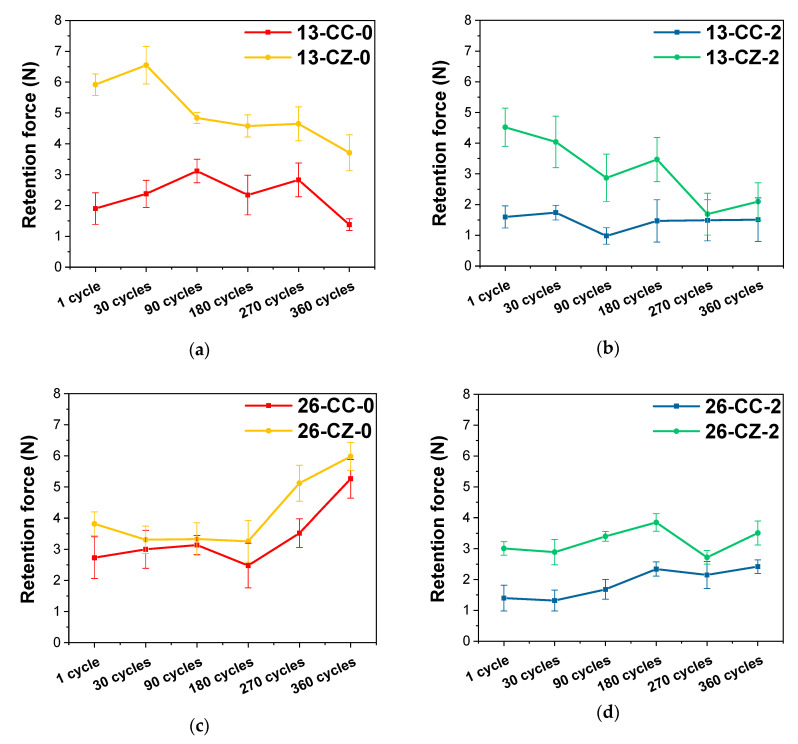
Evolution of the retention forces as a function of the materials used for manufacturing the telescopic system. (**a**) tooth 1.3—taper angle 0°; (**b**) tooth 1.3—taper angle 2°; (**c**) tooth 2.6—taper angle 0° and (**d**) tooth 2.6—taper angle 2°.

From the values recorded after the mechanical tests, it can be observed that the initial values of the retention force of the telescopic system manufactured for tooth 1.3 at a 2° angle are smaller than in the case of 0°, irrespective of the material from which the primary crown was manufactured (Figure 6). The same observation was made in the case of the telescopic tooth system 2.6 

From the visible differences between the values obtained in the mechanical tests, it appears that the shape of the telescopic system (anatomically dictated by the shape of the tooth) also plays an important role in its performance over time, the systems being affected in different ways of wear. 

For tooth systems 1.3, the forces recorded after 360 cycles to simulate use for one year were inferior to those initially recorded after the first detach of the crowns; while tooth 2.6 had the opposite performance, the forces have increased with the passage of time. This is due to their shape, because in the case of the tooth 2.6, the walls’ contact surfaces are much larger, and the friction points through which the crown coupling is made, in time, shifted into friction surfaces, requiring a greater force to separate them, unlike the case of the crowns of tooth 1.3, where the contact surfaces are smaller. The taper angle of the primary crown walls also affected the retention forces, and higher values were required for detaching the systems with an angle of 0° than for 2°, both initially and after 360 cycles.

**Figure 7:**
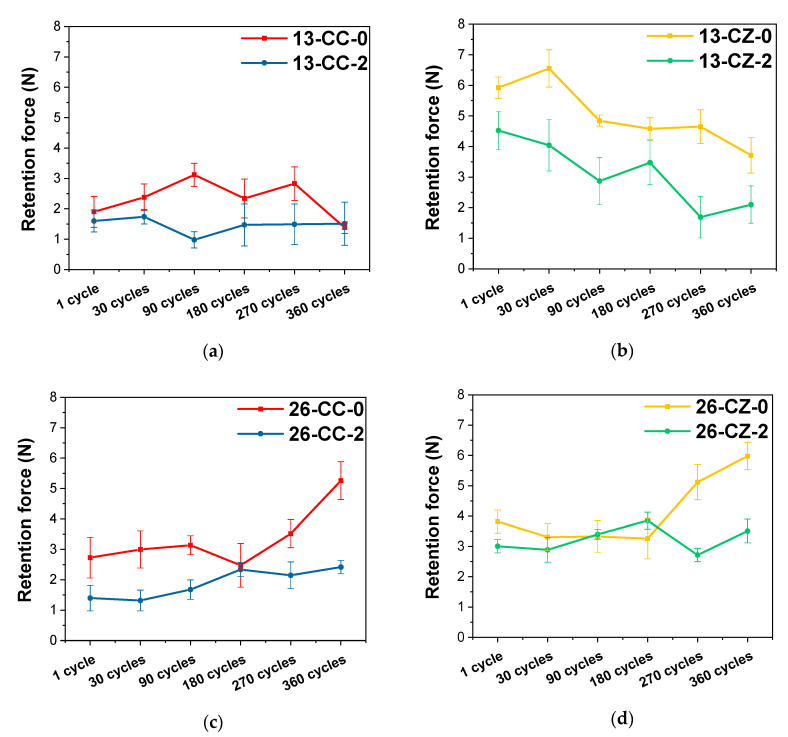
Evolution of the retention forces as a function of the tapper angle used in the manufacturing the telescopic system. (**a**) tooth 1.3—Co–Cr alloy for both crowns; (**b**) tooth 1.3—Co-Cr alloy for secondary crown and ZrO_2_ for primary crown; (**c**) tooth 2.6—Co–Cr alloy for both crowns and (**d**) tooth 2.6 —Co–Cr alloy for secondary crown and ZrO_2_ for primary crown.

One of the main aspects that the present study has highlighted, is that, regardless of the tooth position on the arcades or taper angle, it is obvious that the telescopic system made from ZrO_2_ for the primary crown and the Co–Cr alloy for the secondary crown registered the highest values, indicating an enhanced behavior in terms of retention forces (Figure 7).

In terms of crown materials, zirconia primary crown systems have registered higher forces than those with both Co–Cr alloy crowns, suggesting that they are more feasible for the use of telescopic systems. The values of the forces recorded for the Co–Cr system are largely within the range of 1–3 N, which are rather low values, that could cause potential problems during use. This could be explained through the usage of a hard and wear resistant primary crown material (ZrO_2_) against a less hard secondary crown material (Co–Cr), as in the metal-ceramic biomaterials coupling, which may be considered advantageous.

If we also take into consideration the taper angle of the telescopic system, it is obvious that the retention force has increased when the angle decreased in all samples. This finding is in strong correlation with other literature studies [61,65,66,67]. According to Langer [7,54], in the double-crown systems with a taper angle, the compressive stress is induced by the primary crown, which acts like a wedge due to the occlusal force. A higher angle between the primary and secondary crown leads to a lower compressive force.

The values of the forces for tooth 1.3 decreases after the 360 cycles, while for tooth 2.6, they had grown regardless of the taper angle at which the telescopic system was designed. The variations in the retention forces recorded during the tests are probably due to friction wear products, the formation of new contact micro-zones and/or the modification/disappearance of existing ones between the components of the telescopic system. 

### 3.3. Identification of Contact Areas between the Primary and Secondary Crowns

After the samples were subjected to mechanical tests to evaluate the retention forces by mechanical traction tests, they were macroscopically analyzed (visual inspection of the crown surfaces: naked eye and using magnifying glass/×3.5) in order to study the wear traces that appeared as a result of the cycles performed. The surfaces were compared with those obtained initially to observe the differences arising from the wear process. 

Following the first set of 30 cycles that is the equivalent of 1 month, very few differences occurred, and only small scratches were observed. Wear traces were not identified even on the second set of cycles for the simulation of 3 months (90 cycles). Commencing with 180 cycles (6 months simulation), traces left on the crown surface start being more obvious. The same phenomenon was encountered for the samples tested for 270 cycles (9 months simulation). 

**Figure 8:**
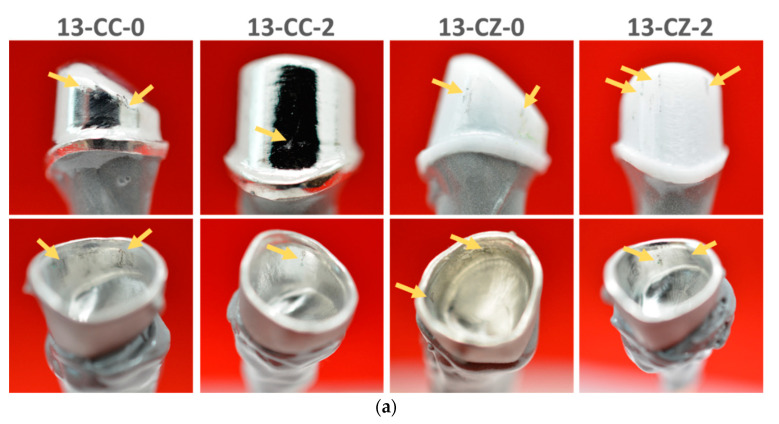

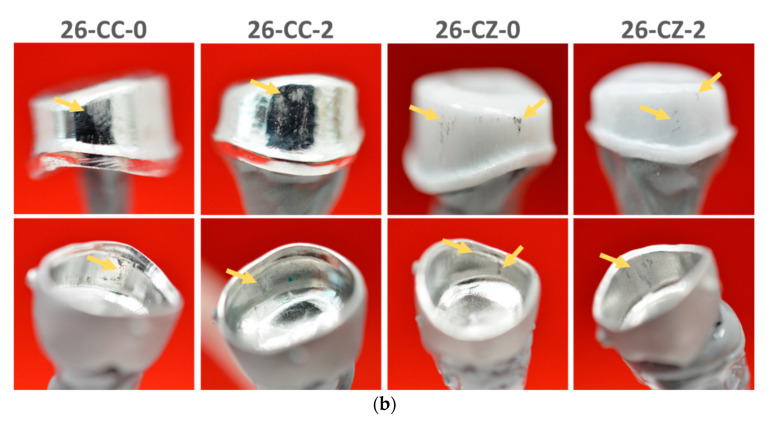
Macroscopic aspect of the telescopic system after the mechanical tests for 360 cycles (the arrows indicate the wear traces). (**a**) tooth 1.3 with taper angle of 0° and 2°, respectively and (**b**) tooth 2.6 with taper angle of 0° and 2°, respectively.

The macroscopic images of the crowns surface of the telescopic systems subjected to mechanical tests, simulating 360 cycles showed defects in the form of scratches on large surfaces and with depths greater than those previously exposed by the wear process. Nevertheless, all wear traces identified on the surfaces examined are due to the friction between the primary and secondary crowns of the telescopic system and are indicated by arrows in Figure 8. Wear traces differ not only in the number of cycles but also as a function of the material. Telescopic systems with zirconia primary crowns generally presented fewer and smaller defects, often two or three, and were positioned in the upper surface of the contact walls, compared to the telescopic systems composed of Co–Cr, where scratches were visible on the entire surface of the contact walls. These traces are due to the different level of material hardness used to manufacture the primary and secondary crowns, components of the telescoping system.

Thus, it can be said that zirconia primary crown telescopes presented more advantages, compared to Co–Cr alloy primary crowns, especially in terms of the retention forces evolution, but also of the wear and friction suffered during the period of 360 cycles test (approximately one year of use). From a clinical point of view, the present study can open new perspectives in terms of biomaterials that can be used for manufacturing the telescopic systems, and which taper angle can offer an overall higher and more adequate behavior.

## 4. Conclusions

The results obtained in this study indicate that the retention forces corresponding to the telescopic systems in which the primary crown is made of zirconia registered the highest values, comparing to the ones corresponding to the telescopic systems with Co–Cr primary crowns. Furthermore, the evolution of the retention forces and wear resistance during the 360 cycles (representing the equivalent of one-year usage of the system) is favorable for the zirconia telescopic primary crowns.

Moreover, by using zirconia-based ceramic as the material of choice for the development of primary crowns, some limitations, such as unaesthetic aspects that are usually associated with metallic materials [68], can be overcome. Thus, by using a telescopic system made from zirconia as primary crown and Co–Cr as material for the secondary crown several enhancements can be brought in both aesthetic and functionality terms. 

All the telescopic systems studied highlighted that by using a taper angle of 2°, the retention force decreases, irrespective of the materials used for the fabrication of the primary crown, indicating that by using a taper angle of 0°, which is known to be ideal, a more efficient and reliable prosthesis can be developed. Even though the ceramic–metallic telescopic system exhibited the best retention, it is worth mentioning that all the double crowns evaluated have registered values between 2–7 N, indicating that they are suitable for clinical use. 

Nevertheless, further investigations of these aspects require intraoral follow-up studies, in terms of retention force evaluation combined with monitoring of the patient level of satisfaction. 

## Figures and Tables

**Table 1:** Codification of the obtained telescopic systems.

Tooth Type	Telescopic System—Materials		Taper Angle	Samples Codification
	Secondary Crown	Primary Crown		
Canine (tooth 1.3)	Co–Cr	Co–Cr	0°	13-CC-0
			2°	13-CC-2
		ZrO_2_	0°	13-CZ-0
			2°	13-CZ-2
1st molar (tooth 2.6)	Co–Cr	Co–Cr	0°	26-CC-0
			2°	26-CC-2
		ZrO_2_	0°	26-CZ-0
			2°	26-CZ-2

**Table 2:** Retention forces registered for the telescopic systems.

Telescopic System	Retention Forces [N]					
	1 Cycle	30 Cycles	90 Cycles	180 Cycles	270 Cycles	360 Cycles
13-CC-0°	1.90 ± 0.51	2.38 ± 0.44	3.12 ± 0.38	2.34 ± 0.64	2.83 ± 0.55	1.38 ± 0.19
13-CC-2°	1.60 ± 0.36	1.74 ± 0.24	0.98 ± 0.27	1.47 ± 0.69	1.49 ± 0.67	1.51 ± 0.71
13-CZ-0°	5.92 ± 0.35	6.55 ± 0.61	4.84 ± 0.18	4.58 ± 0.36	4.65 ± 0.55	3.71 ± 0.58
13-CZ-2°	4.52 ± 0.62	4.04 ± 0.84	2.87 ± 0.77	3.47 ± 0.72	1.69 ± 0.68	2.10 ± 0.61
26-CC-0°	2.73 ± 0.67	3.00 ± 0.61	3.14 ± 0.31	2.48 ± 0.72	5.12 ± 0.58	5.26 ± 0.62
26-CC-2°	1.40 ± 0.42	1.32 ± 0.34	1.68 ± 0.32	2.34 ± 0.23	2.15 ± 0.44	2.42 ± 0.22
26-CZ-0°	3.82 ± 0.38	3.31 ± 0.44	3.33 ± 0.52	3.26 ± 0.67	3.52 ± 0.46	5.98 ± 0.45
26-CZ-2°	3.01 ± 0.22	2.89 ± 0.41	3.40 ± 0.16	3.85 ± 0.28	2.72 ± 0.22	3.51 ± 0.39
